# Estimating apple tree canopy chlorophyll content based on Sentinel-2A remote sensing imaging

**DOI:** 10.1038/s41598-018-21963-0

**Published:** 2018-02-28

**Authors:** Cheng Li, Xicun Zhu, Yu Wei, Shujing Cao, Xiaoyan Guo, Xinyang Yu, Chunyan Chang

**Affiliations:** 1 College of Resources and Environment, Shandong Agricultural University, Tai’an, 271018 China; 2 Key Laboratory of Agricultural Ecology and Environment, Shandong Agricultural University, Tai’an, 271018 China

## Abstract

The remote sensing technology provides a new means for the determination of chlorophyll content in apple trees that includes a rapid analysis, low cost and large monitoring area. The Back-Propagation Neural Network (BPNN) and the Supported Vector Machine Regression (SVMR) methods were both frequently used method to construct estimation model based on remote sensing imaging. The aim of this study was to find out which estimation model of apple tree canopy chlorophyll content based on the vegetation indices constructed with visible, red edge and near-infrared bands of the sensor of Sentinel-2 was more accurate and stabler. The results were as follows: The calibration set coefficient of determination (R^2^) value of 0.729 and validation set R^2^ value of 0.667 of the model using the SVMR method based on the vegetation indices (NDVI_green_ + NDVI_red_ + NDVI_re_) were higher than those of the model using the BPNN method by 8.2% and 11.0%, respectively. The calibration set root mean square error (RMSE) of 0.159 and validation set RMSE of 0.178 of the model using the SVMR method based on the vegetation indices (NDVI_green_ + NDVI_red_ + NDVI_re_) were lower than those of the model using the BPNN method by 5.9% and 3.8%, respectively.

## Introduction

Chlorophyll is the main carrier of photosynthesis in plants^1^. By monitoring the chlorophyll content of the apple canopy, the photosynthetic capacity, nutrient stress and developmental stage of an apple tree can be detected^2^. This can also indirectly reflect the apple tree nitrogen content, physiological status, etc. In addition, chlorophyll is an important means for assessing the growth of apple trees as well as pests and diseases, crop yield and crop maturity^3,4^. Traditional chlorophyll content analysis in the laboratory and the need for field sampling is expensive in terms of manpower, materials and time, and cannot meet the real-time, large area monitoring and rapid management of apple trees. The remote sensing technology provides a new means for the determination of chlorophyll content in apple trees that includes a rapid analysis, low cost and large monitoring area. Remote sensing technology is based on electromagnetic waves absorbed and reflected by objects to form a spectral absorption and reflection feature in specific locations of the electromagnetic waves. This reflects the composition and structure of an object and remote sensing technology could achieve the goal of detecting the features and properties of the objects^5^. The application of remote sensing technology to the rapid monitoring of chlorophyll content in apple trees is of great significance for guiding the scientific management of apple trees^6,7^.

Many studies have been completed on vegetation monitoring using multispectral satellites. However, due to the limitation of technology, the sensor carried by the multispectral satellite included mainly bands of blue, green, red and near-infrared. With the development of remote sensing technology, the spectral bands of the sensor carried by the satellite has been increased to improve satellite application capabilities, such as Germany’s satellite RapidEye and the United States Satellite Worldview-2. The sensor of Sentinel-2 launched by the European Space Agency (ESA) was added the bands of red edge, which could provide new data to monitor the green plant.

Regarded as one of the most obvious characteristics of green vegetation, “red edge” refers to the transition platform from the strong absorption of red light to the near-infrared multiple scattering of vegetation chlorophyll. When the vegetation is vigorous, the pigment content is high, and the red edge moves to the long wave direction, called “redshift”; otherwise, the red edge moves to the shortwave direction and is called “blueshift”. The red edge was found to be sensitive to the chlorophyll content of the canopy, and the chlorophyll content could be well estimated by red edge^8–10^. Red edge parameters are mainly the red edge position, red edge average reflectivity, the red edge slope, red edge amplitude, ratio of red edge amplitude and minimum amplitude, and red edge area, among others.

Liu Wenya *et al*. analysed the correlation between the spectral reflectance of the canopy and the chlorophyll content during different growth stages and built the estimation model of chlorophyll content with 7 different red edge parameters and a BPNN. The results showed that the estimation model is effective^11^. YAO Fuqi *et al*. analysed the correlation between red edge characteristics and different chlorophyll content (SPAD) of winter wheat in different growth stages, proving the feasibility of using red edge parameters to predict canopy chlorophyll content in winter wheat. Using red edge position and red edge kurtosis, the prediction model of winter wheat SPAD was built during different growth stages. Compared with the red edge position, the red edge kurtosis could improve the accuracy of prediction^12^. Huang Wenjiang used the red edge to predict the chlorophyll content of winter wheat leaves. The estimated value of the model and the true value determination coefficient R^2^ reached 0.7, which could evaluate the growth status of winter wheat and provide an important basis for nutrition diagnosis^13^. Huang Chunyan *et al*. analysed the correlation between the red edge area and red edge slope with the canopy leaf chlorophyll density and leaf area index (LAI) of cotton *Xinlu Early 13* and *Xinlu Early 19*, and this correlation was very significant. Compared with the red edge slope, the red edge area had a better correlation with the canopy leaf chlorophyll density and LAI, and the prediction accuracy of chlorophyll density and LAI was 87.4% and 83.3%, respectively^14^. Jago *et al*. Found that there was a good correlation between the red edge position and chlorophyll accumulation under the topdressing conditions^15^. Using the linear extrapolation method, Cho *et al*. extracted the red edge position from the spectral data. The study shows that the correlation between red edge parameters and chlorophyll reached 0.86 at the wavelength of 680 nm, 694 nm, 724 nm and 760 nm, which reduced the influences of the background spectrum noise^16^. Main *et al*. attempted to analyse the 73 spectral indices, and selected the optimal index to predict chlorophyll content. The results showed that REP-LE (red edge position linear extrapolation) spectral index is better^17^. In the study of chlorophyll content estimation from home and abroad, the red edge parameters correlated well with the chlorophyll content of vegetation. However, the studies discussed above were based on the data measured by the near-earth imaging or non-imaging spectrometer. In the study of vegetation chlorophyll content by multispectral satellites, most of them were limited to the range of the satellite sensor band, and the red edge band related to chlorophyll content was considered less. Studies on leading the red edge band into the vegetation index to construct an estimation model of the fruit tree canopy chlorophyll content has been rarely reported.

In summary, because the red edge band is sensitive to the chlorophyll content, the remote sensing image including the red edge band is the preferred image for estimating the vegetation chlorophyll content at a large-scale rapidly and non-destructively^18^. In this study, the chlorophyll content of the apple canopy was estimated using Sentinel-2A remote sensing image and the measured data of near-earth in the city of Qixia of the Shandong Province. In the hilly part of the study area, the Minnaert model was used to correct the topographic radiation of the remote sensing image after atmospheric correction, and the reflectance of the surface was extracted. Then, based on the spectral index constructed by predecessors, the chlorophyll vegetation index of the apple canopy was selected and constructed by using the Sentinel-2A image bands of blue, green, red, red edge and near-infrared. Finally, the chlorophyll content estimation model of the apple canopy was constructed based on the vegetation index. Comparing the accuracy of different models, the best estimation model was selected.

## Materials and Methods

### The research area

**Figure 1:**
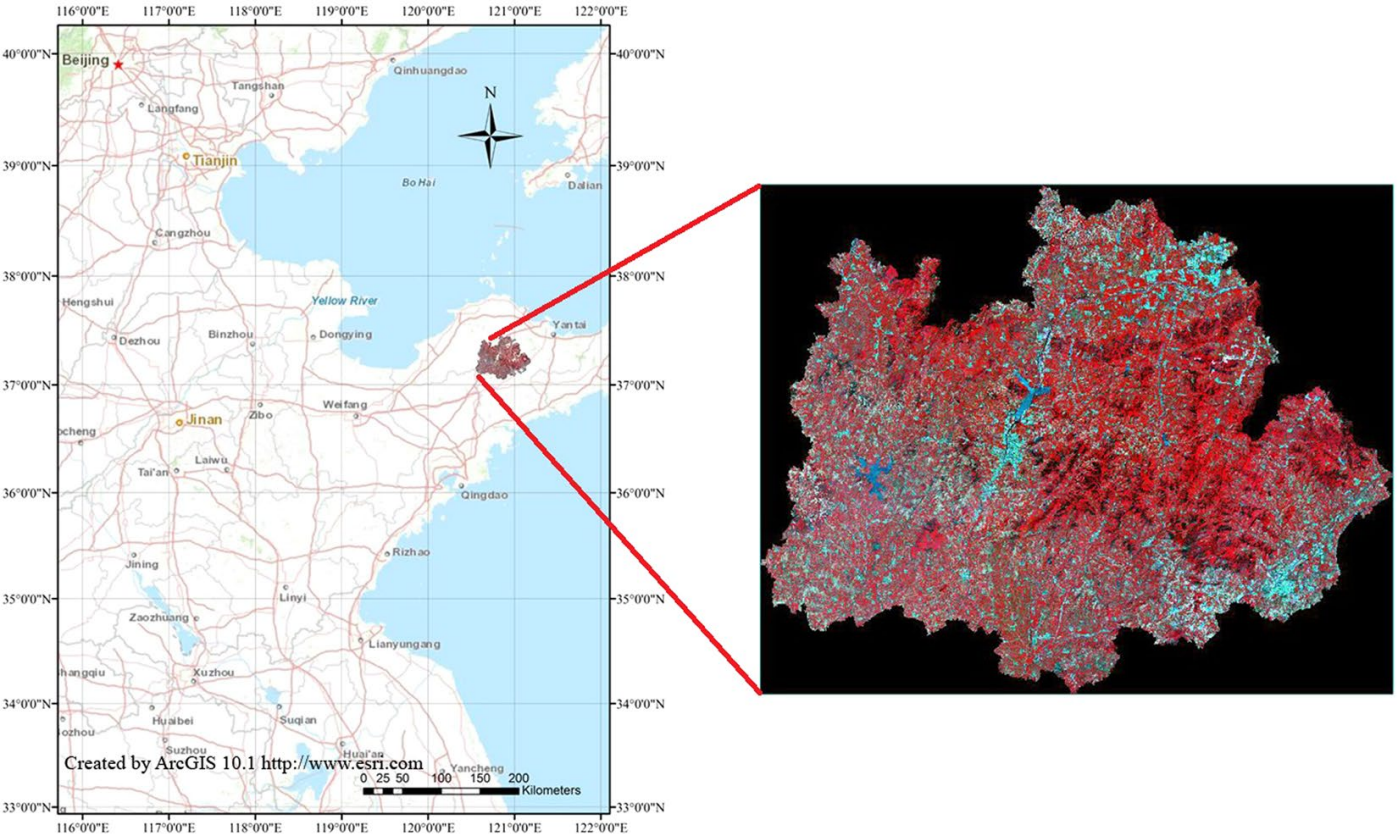
Location of the study area.

The research area is located in the city of Qixia, of the Shandong province, China (120°33′~121°15′E, 37°05′~37°32′N) (e.g., Fig. 1). The area is a warm temperate, monsoonal, semi humid climate, where there is adequate illumination, the annual average temperature is 11.3 °C, and the rainfall is approximately 650 mm. With 72.1% mountain, 21.8% hilly land and 6.1% plain, the study area is rugged. The main soil type is brown soil. The orchard area is up to 4.3 × 10^4^ hm^2^, and the apple annual yield is approximately 1.2 × 10^7^ kg. Because the industrialization, standardization and internationalization level ranking in China is first, the city of Qixia is known as “Apple Capital”.

### Sample collection

On June 16–17, 2016, 52 orchards were randomly selected in 15 towns in Qixia. The sample points were distributed evenly in the orchard, away from other obvious features (roads, canals, etc.), and the coordinates of the central sample point were measured by GPS. Three trees in each orchard were randomly selected to represent the average growth of fruit trees in the orchard. Twenty-four healthy, non-destructive and regular leaves were collected from the east, west, south, and north directions and from the upper, middle, and lower layers of the canopy of the apple tree. The leaves were placed into a freshness protection package, and then the packages were placed into an ice box. The chlorophyll content was measured by *UV762 Ultraviolet-Visible Spectrophotometer* in the laboratory.

The canopy spectrum data were measured by the *ASD FieldSpec 4* portable surface features spectrometer, which has 2151 bands ranging from 350 nm to 2500 nm. The sampling interval of the bands from 350 nm to 1000 nm is 1.4 nm, and the spectral resolution in these bands is 3 nm. The sampling interval of the bands from 1001 nm to 2500 nm is 2 nm, and the spectral resolution of these bands is 8 nm. A clear, calm weather day was chosen for measuring the spectra. Measurements were taken between 10:00–14:00 when the solar elevation angle was greater than 45° to reduce the error due to the change of illumination. The spectrometer was corrected by the standard white plate before determination. Measurement of the apple tree canopy spectral data was as follows. A spectrometer external 5 m jump fiber was used, with a probe that was vertical downward and its view angle was 25°. Then, according to the apple tree canopy size, the distance between the probe and the canopy was adjusted to ensure the probe field covered the entire canopy. Each canopy was measured 10 times, and the arithmetic mean of the 3 canopies at the sampling point was used as the spectral reflectance data of this sample point.

### Sentinel-2A image data

Satellite Sentinel-2A was launched by the ESA on June 23, 2015, equipped with a new high-resolution multispectral imager (MSI). The MSI imager has 13 bands (e.g., Table 1). It contains three spatial bands with a spatial resolution of 20 m. It is the first civil, optically observable satellite with three “red edge” bands, which provides more band selection for the dynamic monitoring of chlorophyll content in vegetation. According to the time of sampling on the ground, the remote sensing image for June 16, 2016 was selected. The cloudiness was less, and the quality was better. The acquired Sentinel-2A multispectral image data type is Level-1C, which is the reflectivity data for the TOA that has been geometrically corrected.

**Table 1:** Band parameters of the Sentinel-2A MSI imager.

Band	Name	Wavelength range/μm	Resolution/m
Band 1	Coastal aerosol	0.433–0.453	60
Band 2	Blue	0.458–0.523	10
Band 3	Green	0.543–0.578	10
Band 4	Red	0.650–0.680	10
Band 5	Vegetation red edge	0.698–0.713	20
Band 6	Vegetation red edge	0.733–0.748	20
Band 7	Vegetation red edge	0.773–0.793	20
Band 8	Near-infrared	0.785–0.900	10
Band 8 A	Near-infrared narrow	0.855–0.875	20
Band 9	Water vapour	0.935–0.955	60
Band 10	Shortwave infrared-Cirrus	1.360–1.390	60
Band 11	Shortwave infrared	1.565–1.655	20
Band 12	Shortwave infrared	2.100–2.280	20

### Image pre-processing

The acquired Sentinel-2A image data is the reflectance of the TOA, and the reflectance of the BOA and can be obtained after atmospheric correction using the SNAP software provided by ESA. As seen from Table 1, the spatial resolution of each band of the Sentinel-2A data is not consistent, and the bands after atmospheric correction were resampled to 10 m using the nearest neighbour interpolation method. In the research area, there is a difference in the effective illumination received because of the terrain variation. The spectral information of the surface is disturbed by the phenomenon of different objects which have the same spectrum or the same objects that have a different spectrum. The precision of the quantitative estimation of the surface parameters is affected^19^. The Minnaert model^20–22^ was used to perform topographic correction of the image to eliminate or reduce the reflectance difference caused by the topography in the study area.

### Constructing and selecting the vegetation index

The vegetation index is a combination of the relevant spectral signals, which are two or more bands of multispectral or hyperspectral remote sensing data combined through a certain mathematical transformation^23–25^. Among them, the “red edge”, which reflects the change of reflectivity from the low value band of chlorophyll red absorption to the high value band of canopy scattering, is the most obvious feature of the green vegetation spectral curve^8^. This change is due to the scattering of leaves and canopy^7^. Chlorophyll forms strong absorption peaks in blue and red bands, absorption valleys in the green band and very little absorption in the near-infrared band. The spectral responses of chlorophyll content in the canopy of green vegetation are different from those in blue, green, red, red edge and near-infrared bands, which is the physical basis for the remote sensing monitoring of vegetation^26^. By considering the spectral characteristics of the green vegetation and the bands of the Sentinel-2A image, using the principle of ratio vegetation index (RVI), chlorophyll index (CI) and normalized differential vegetation index (NDVI) for reference, the blue band, green band, red band, red edge band and near-infrared band were leading to the vegetation index and its transformation in the form of the vegetation index constructed, as shown in Table 2.

**Table 2:** The vegetation indices for monitoring of chlorophyll content.

Plant indices	Calculation formula	Plant indices	Calculation formula
RVI_blue_	ρ_3_/ρ_2_	CI_red_	ρ_8_/ρ_4_ − 1
RVI_green_	ρ_3_/ρ_4_	CI_re_	ρ_8A_/ρ_7_ − 1
RVI_red_	ρ_8_/ρ_4_	NDVI_blue_	(ρ_3_ − ρ_2_)/(ρ_3_ + ρ_2_)
RVI_re_	ρ_8A_/ρ_7_	NDVI_green_	(ρ_3_ − ρ_4_)/(ρ_3_ + ρ_4_)
CI_blue_	ρ_8_/ρ_2_ − 1	NDVI_red_	(ρ_8_ − ρ_4_)/(ρ_8_ + ρ_4_)
CI_green_	ρ_8_/ρ_3_ − 1	NDVI_re_	(ρ_8A_ − ρ_7_)/(ρ_8A_ + ρ_7_)

Note: ρ_2_, ρ_3_, ρ_4_, ρ_7_, ρ_8_, ρ_8A_ represent the surface reflectance of blue band 2, green band 3, red band 4, red edge band 7, band 8 and near-infrared band 8A, respectively.

Twelve vegetation indices based on Sentinel-2A band were constructed to analyse the correlation with the canopy chlorophyll content. Fully considering the spectral information but also to avoid the redundancy of data, the correlation of the bands in the series 1 vegetation indices (RVI_blue_, RVI_green_, RVI_red_ and RVI_re_), series 2 vegetation indices (CI_blue_, CI_green_, CI_red_ and CI_re_) and series 3 vegetation indices (NDVI_blue_, NDVI_green_, NDVI_red_ and NDVI_re_) were analysed. The correlation between the bands can reflect the independent and redundant information situation of bands. The greater the correlation, the stronger the consistency of the information between the bands and the greater the amount of information redundancy; however, the amount of information redundancy was lower. The correlation between bands was measured by the correlation coefficients between bands, and the formula is as follows:

1 $${R}_{ij}=\frac{{{\rm{\sigma }}}_{{\rm{ij}}}^{2}}{{\sigma }_{{\rm{ii}}}\times {\sigma }_{{\rm{jj}}}},$$

In formula 1, *R*_*ij*_ is the correlation coefficient of the two bands I and J; ${{\rm{\sigma }}}_{{\rm{ij}}}^{2}$ is the covariance of the two bands I and J; and σ_ii_ and σ_jj_ are the standard deviations of the two bands I and j, respectively.

### Building and testing the of estimation model

To build the estimation model of apple tree canopy chlorophyll content, 39 samples were chosen from 52 samples randomly collected in the field and were used to build the estimation model. The other 13 samples were used for the model test. The BPNN and SVMR method were used to build the estimation model of apple tree canopy chlorophyll content. The BPNN model, using the gradient descent method widely used in optimization problems, is a method to translate the input-output problem to a nonlinear optimization problem. In addition, it is highly nonlinear to map from input to output^27^, thus revealing the nonlinear relationship among samples. This method has been widely used in hyperspectral quantitative estimation. Based on the principle of structural risk minimization, the SVMR method overcomes the problems of over learning and falling into a local minimum in the traditional statistical methods, and it has a very strong generalization capacity^28^. The kernel function method is used for mapping the higher dimensional space without increasing the computational complexity, and overcoming the problem of dimensionality. The SVMR method is widely used in statistical classification and regression analysis; the mathematical form is concise and suitable for small sample analysis. The determinant coefficient (R^2^) and the root mean square error (RMSE) were used to test the model. A higher R^2^ indicated that the model was more stable, and a lower RMSE indicated great model accuracy^29^. The formulas of R^2^ and EMSE are as follows:

2 $${R}^{2}=1-\frac{{\sum }_{i=1}{({y}_{i}-{\hat{y}}_{i})}^{2}}{{\sum }_{i=1}{({y}_{i}-{\bar{y}}_{i})}^{2}},$$

3 $$RMSE=\sqrt{\frac{{\sum }_{i=1}^{n}{({y}_{i}-{\hat{y}}_{i})}^{2}}{n}},$$

## Results and Discussion

### Results of image correction and discussion

#### Results of atmospheric correction and discussion

**Figure 2:**
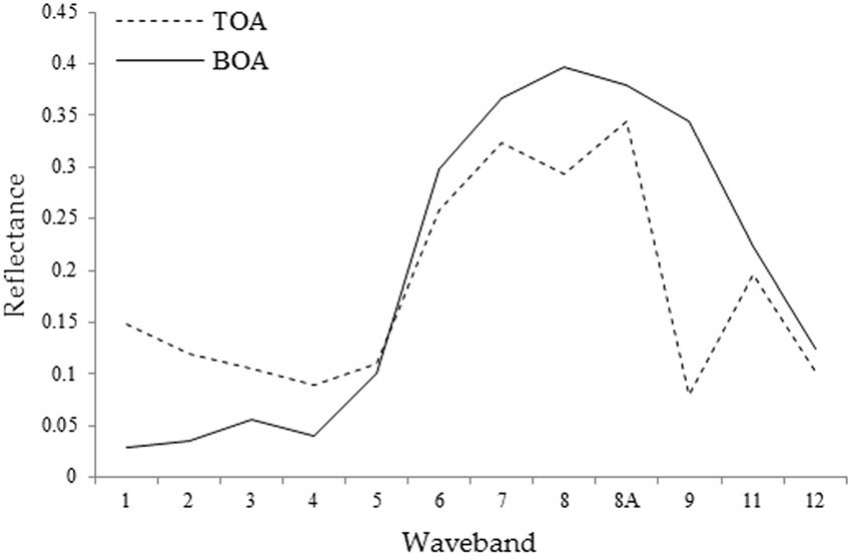
Comparison of vegetation reflectance curves between the original image and atmospheric correction image.

Comparing the reflectance of the top of the atmosphere (TOA) with the reflectivity of the bottom of the atmospheric (BOA), which was corrected using atmospheric correction, the vegetation spectrum after atmospheric correction shows the typical trend of the green vegetation spectrum (e.g., Fig. 2). The 0.49 μm blue light and 0.665 μm red light showed two absorption bands which was the canopy of chlorophyll absorption of light radiation photosynthesis forming two reflection troughs. There was a small reflection peak at the 0.56 μm green light, which represented the strong reflection effect of chlorophyll on green light in green vegetation. In the range of 0.705–0.783 μm, the red edge was obvious, and a higher reflection platform was formed between the bands 8 and 9, which was formed by the scattering of the near-infrared. The influence of the atmosphere on the remote sensing image was weakened by using the atmospheric correction model, and the trend of vegetation spectral curve was restored well^30^.

#### Results of the Minnaert model correction and discussion

**Figure 3:**
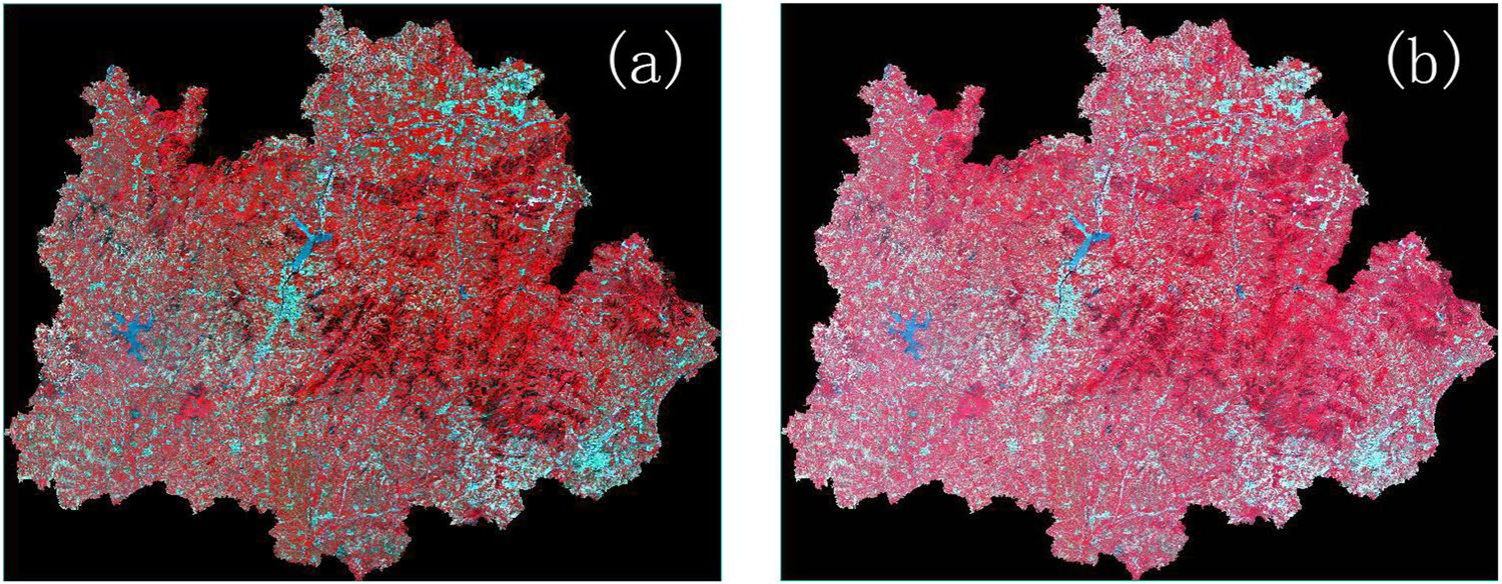
Image (**a**) is the image before Minnaert correction and image (**b**) is the image corrected by the Minnaert model.

**Figure 4:**
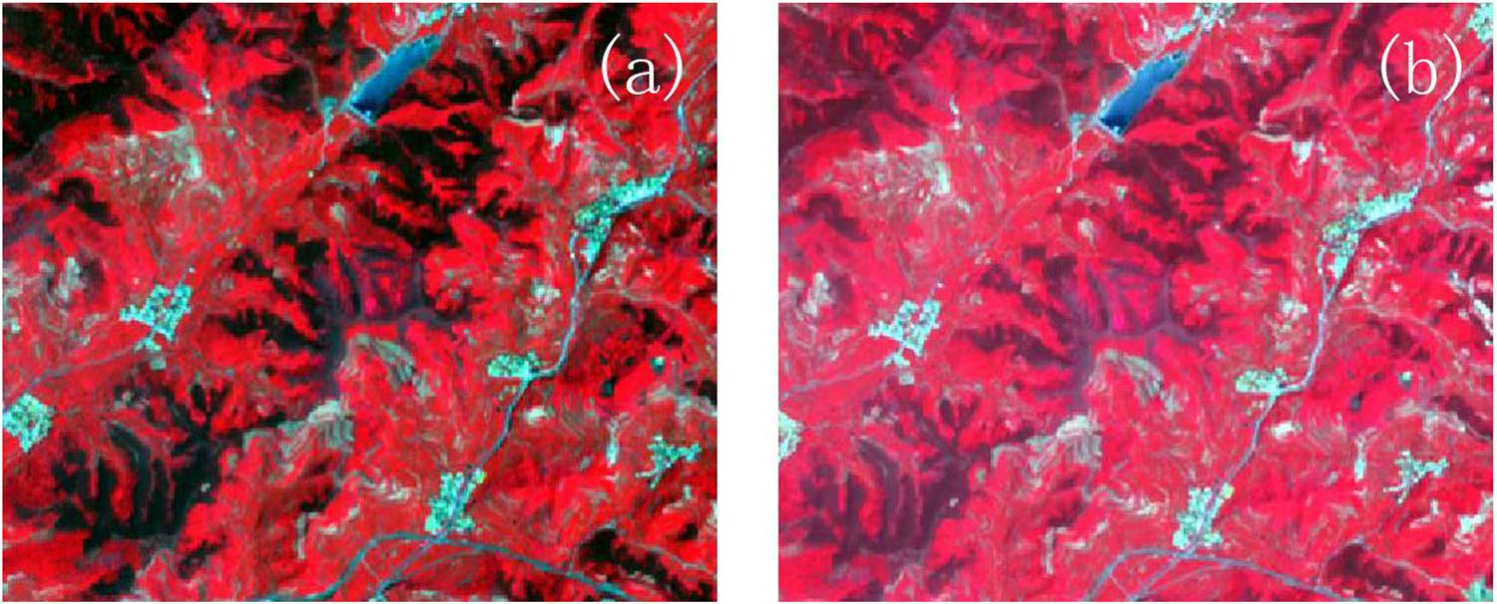
Image (**a**) is the local image before Minnaert correction, and image (**b**) is the local image corrected by Minnaert model.

Comparing the atmospheric-corrected image with the image after the Minnaert model correction, the terrain effect was weakened after the Minnaert model correction (e.g., Fig. 3). To see the details of the corrected image transformation, the same region of the images before and after the Minnaert model correction, including sunny and shady slopes, were selected to comparison. The result of the false colour composite is shown in Fig. 4. The surface information of the shaded area was restored to varying degrees after the Minnaert model correction.

### Extraction of the surface albedo and precision analysis

According to the GPS coordinates of the field measurement, the sample points were located in the Sentinel-2A image with the error controlled in one pixel. If the GPS coordinates ware located at the boundary of two or more pixels, the average of the spectral reflectance of the neighboring pixels was deemed to be the reflectance of the sample point. If the GPS coordinates ware located at the central position of the pixel, the spectral reflectance of the pixel was deemed to be the reflectance of the sample point. The apparent reflectance and the surface albedo of the sample area were obtained. The average value of each band of the field measured reflectance, apparent reflectance and the surface albedo were compared and analysed, and the relative error was calculated (e.g., Table 3).

**Table 3:** Comparison of relative errors of Sentinel-2A reflectivity.

Relative error	Band 2	Band 3	Band 4	Band 5	Band 6	Band 7	Band 8	Band 8 A
Relative error of TOA	336.5%	163.3%	234.3%	103.8%	48.1%	43.9%	38.7%	44.2%
Relative error of BOA	29.5%	19.4%	32.3%	16.1%	11.1%	10.6%	9.7%	9.0%

Note: the relative errors of TOA and BOA were the relative errors of the measured reflectance with TOA and BOA, respectively.

After atmospheric and topographic corrections, the relative errors between the reflectance of bands 2–8 A and the field measured reflectance of the canopy were 29.5%, 19.4%, 32.3%, 16.1%, 11.1%, 10.6%, 9.7% and 9.0%. This result shows that the truer canopy spectrum can be obtained by various means of image processing, which provides an accurate guarantee for subsequent analysis.

### Construction and screening of vegetation index

The Sentinel-2A image contained three red edge bands, including bands 5 (0.705 μm), 6 (0.740 μm) and 7 (0.783 μm). The correlation between the canopy chlorophyll content and the surface albedo of the three bands was analysed. The correlation coefficients between the canopy chlorophyll content and the surface albedo of the three red edge bands were 0.334, 0.384 and 0.401. The correlation coefficient between the canopy chlorophyll content and the surface albedo of band 7 was higher than that between the canopy chlorophyll content and the surface albedo of the other bands. Therefore, band 7 with a centre wavelength of 0.783 μm was chosen as the red edge band in the vegetation index calculation. The vegetation index constructed in combination with the Sentinel-2A images is shown in Table 2. Three series of vegetation indices were constructed for series 1 (RVI_blue_, RVI_green_, RVI_red_ and RVI_re_), series 2 (CI_blue_, CI_green_, CI_red_ and CI_re_) and series 3 (NDVI_blue_, NDVI_green_, NDVI_red_ and NDVI_re_), respectively.

In series 1, the correlation coefficients between RVI_blue_ and RVI_green_, RVI_red_, and RVI_re_ were 0.339, 0.367 and −0.103, respectively; the correlation coefficients between RVI_green_ and RVI_red_, and RVI_re_ were 0.978 and −0.319, respectively; the correlation coefficient between RVI_red_ and RVI_re_ was −0.331. The correlation coefficient between RVI_green_ and RVI_red_ was too high, so only one was considered for construction of the estimation model. Eventually, RVI_blue_, RVI_red_ and RVI_re_ were selected for series 1 because the correlation coefficient between the canopy chlorophyll content and RVI_red_ was −0.331 higher than that between the canopy chlorophyll content and RVI_green_ (e.g., Table 4).

**Table 4:** The correlation coefficient of plant parameters and chlorophyll content.

Plant indices	Correlation coefficient	Plant indices	Correlation coefficient
RVI_blue_	−0.433**	CI_red_	−0.376**
RVI_green_	−0.304*	CI_re_	0.558**
RVI_red_	−0.331*	NDVI_blue_	−0.397**
RVI_re_	0.546**	NDVI_green_	0.469**
CI_blue_	−0.391**	NDVI_red_	−0.339*
CI_green_	0.314*	NDVI_re_	0.525**

Note: **significant at 0.01 level; *significant at 0.05 level.

In series 2, the correlation coefficients between CI_blue_ and CI_green_, CI_red_, and CI_re_ were 0.429, 0.492 and −0.223, respectively; the correlation coefficients between CI_green_ and CI_red_, and CI_re_ were 0.914 and −0.353, respectively; the correlation coefficient between CI_red_ and CI_re_ was −0.231. The correlation coefficient between CI_green_ and CI_red_ was too high, so only one was considered for construction of the estimation model. Eventually, CI_blue_, CI_red_ and CI_re_ were selected for series 2 because the correlation coefficient between the canopy chlorophyll content and CI_red_ was −0.376 higher than that between the canopy chlorophyll content and CI_green_ (e.g., Table 4).

In series 3, the correlation coefficients between NDVI_blue_ and NDVI_green_, NDVI_red_, and NDVI_re_ were 0.817, −0.387 and 0.091, respectively; the correlation coefficients between NDVI_green_ and NDVI_red_, and NDVI_re_ were 0.287 and 0.027, respectively; the correlation coefficient between NDVI_red_ and NDVI_re_ was 0.257. The correlation coefficient between NDVI_blue_ and NDVI_green_ was too high, so only one was considered for construction of the estimation model. Eventually, NDVI_green_, NDVI_red_ and NDVI_re_ were selected for series 3, because the correlation coefficient between the canopy chlorophyll content and NDVI_green_ was 0.469 higher than that between the canopy chlorophyll content and NDVI_blue_ (e.g., Table 4).

From the above information, the correlation between the vegetation index was constructed based on the red edge band of the image from Sentinel-2A. The other vegetation indices in the same series ware minimal. The correlation between the vegetation index constructed based on the red edge band of the image from Sentinel-2A and the canopy chlorophyll content was maximal. With less redundant information, the vegetation index constructed based on the red edge band offers more information to estimate the canopy chlorophyll content, and it was suitable for estimating the canopy chlorophyll content.

### Building and test of the chlorophyll content estimation model of the apple tree canopy

#### Building and testing of the BPNN estimation model

The BPNN models 1, 2 and 3 were built using the chemical method, laboratory-measured chlorophyll content of 39 samples as dependent variables, and the vegetation indices series 1 (RVI_blue_, RVI_red_ and RVI_re_), series 2 (CI_blue_, CI_red_ and CI_re_) and series 3 (NDVI_green_, NDVI_red_ and NDVI_re_) as independent variable, respectively. The DPS statistical software and MATLAB were used to build the BPNN models. In the network consists of three layers, and the input, hidden and output layers have 4, 4 and 1 neurons respectively. Other details are shown in Table 5. The parameters were determined through repeated practical training.

**Table 5:** The BPNN model parameters.

Implicit network layer	Input layer node number	The minimum training rate	The dynamic parameters
1	4	0.1	0.6
Sigmoid parameters	Margin of error	The largest number of iterations	First number of hidden layer nodes
0.9	0.0001	1000	3

As shown in Table 6, the determinant coefficient of model 1 (R_BP1c_^2^) was 0.589, and the root mean square error (RMSE_BP1c_) was 0.178. The determinant coefficient of model 2 (R_BP2c_^2^) was 0.623, and the root mean square error (RMSE_BP2c_) was 0.191. The determinant coefficient of model 3 (R_BP3c_^2^) was 0.674, and the root mean square error (RMSE_BP3c_) was 0.169.

**Table 6:** The BPNN models for estimation of chlorophyll content based on vegetation indices.

Characteristic bands	R_BPc_^2^	RMSE_BPc_	R_BPv_^2^	RMSE_BPv_
RVI_blue_ + RVI_red_ + RVI_re_	0.589	0.178	0.523	0.192
CI_blue_ + CI_red_ + CI_re_	0.623	0.191	0.563	0.205
NDVI_green_ + NDVI_red_ + NDVI_re_	0.674	0.169	0.601	0.185

Note: R_BPc_^2^ was the determination coefficient of the BPNN model; R_BPv_^2^ was the verification determination coefficient of the BPNN model; RMSE_BPc_ was the root mean square error of the BPNN model; and RMSE_BPv_ was the verification root mean square error of the BPNN model.

**Figure 5:**
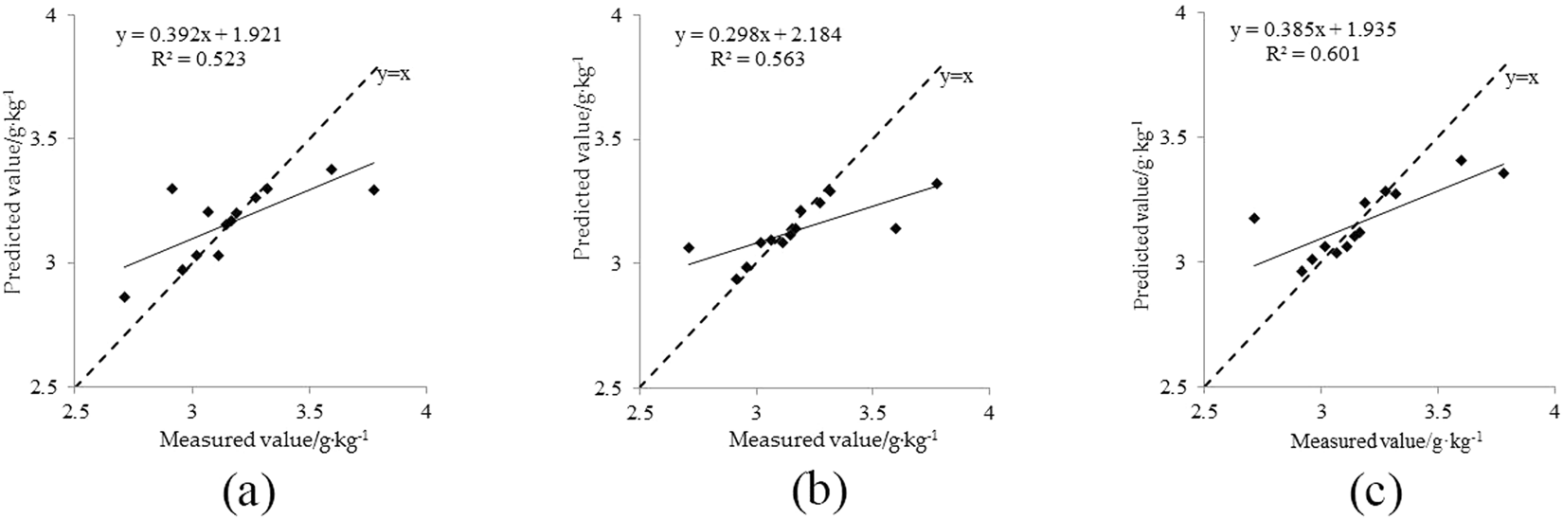
Scatter plots of the measured and predicted values of validation with BPNN 1(**a**), 2(**b**) and 3(**c**) based on vegetation indices.

The surface albedo and chlorophyll content of 13 samples were used to test the model. As shown in Table 6, the verification determinant coefficient of model 1 (R_BP1v_^2^) was 0.523, and the verification root mean square error (RMSE_BP1v_) was 0.192. The verification determinant coefficient of model 2 (R_BP2v_^2^) was 0.563, and the verification root mean square error (RMSE_BP2v_) was 0.205. The verification determinant coefficient of model 3 (R_BP3v_^2^) was 0.601, and the verification root mean square error (RMSE_BP3v_) was 0.185. At the same time, the predicted values of the chlorophyll content of the canopy for the tested samples were compared 1:1 with the measured values (e.g., Fig. 5). The determination coefficient for building and testing of BPNN model 3 based on the vegetation indices series 3 (NDVI_green_, NDVI_red_ and NDVI_re_) was maximal, and its RMSE was minimal. It indicates that model 3 has a better self-estimation and these estimation abilities and can be used for the quantitative estimation of the apple canopy chlorophyll content.

#### Building and testing of the SVMR estimation model

The SVMR model 1, 2 and 3 built using the chemical method, laboratory-measured chlorophyll content of 39 samples as dependent variables, and the vegetation indices series 1 (RVI_blue_, RVI_red_ and RVI_re_), series 2 (CI_blue_, CI_red_ and CI_re_) and series 3 (NDVI_green_, NDVI_red_ and NDVI_re_) as independent variables, respectively. The DPS statistical software was used to build the SVMR model. The model parameters were determined through parameter optimization, model regression and verification, and repeated analysis and comparison. The details are shown in Table 7.

**Table 7:** SVMR model parameters.

Degree	Gamma	Coef0	Nu	Epsilon	Cashesize	Cost	Shrinking	Prob	P
3	0.5	0.001	0.5	0.001	100	1	1	1	0.01

Note: Degree: set degree in kernel function; Gamma: set gamma in kernel function; Coef0: set coef0 in kernel function; Nu: set the parameter nu of nu-SVC, one-class SVM, and nu-SVR; Epsilon: set tolerance of termination criterion; Cashesize: set cache memory size in MB; Cost: set the parameter C of C-SVC, epsilon-SVR, and nu-SVR; Shrinking: whether to use the shrinking heuristics, 0 or 1; Prob: whether to train a SVR model for probability estimates, 0 or 1; P:set the epsilon in loss function of epsilon-SVR.

As shown in Table 8, the determinant coefficient of model 1 (R_SVMR1c_^2^) was 0.627, and the root mean square error (RMSE_SVMR1c_) was 0.183. The determinant coefficient of model 2 (R_SVMR2c_^2^) was 0.663, and the root mean square error (RMSE_SVMR2c_) was 0.179. The determinant coefficient of model 3 (R_SVMR3c_^2^) was 0.729, and the root mean square error (RMSE_SVMR3c_) was 0.159.

**Table 8:** The SVMR models for estimation of chlorophyll content based on vegetation indices.

Characteristic bands	R_SVMRc_^2^	RMSE_SVMRc_	R_SVMRv_^2^	RMSE_SVMRv_
RVI_blue_ + RVI_red_ + RVI_re_	0.627	0.183	0.559	0.197
CI_blue_ + CI_red_ + CI_re_	0.663	0.179	0.577	0.194
NDVI_green_ + NDVI_red_ + NDVI_re_	0.729	0.159	0.667	0.178

Note: R_SVMRc_^2^ was the determination coefficient of the SVMR model; R_SVMRv_^2^ was the verification determination coefficient of the SVMR model; RMSE_SVMRc_ was the root mean square error of the SVMR model; RMSE_SVMRv_ was the verification root mean square error of the SVMR model.

**Figure 6:**
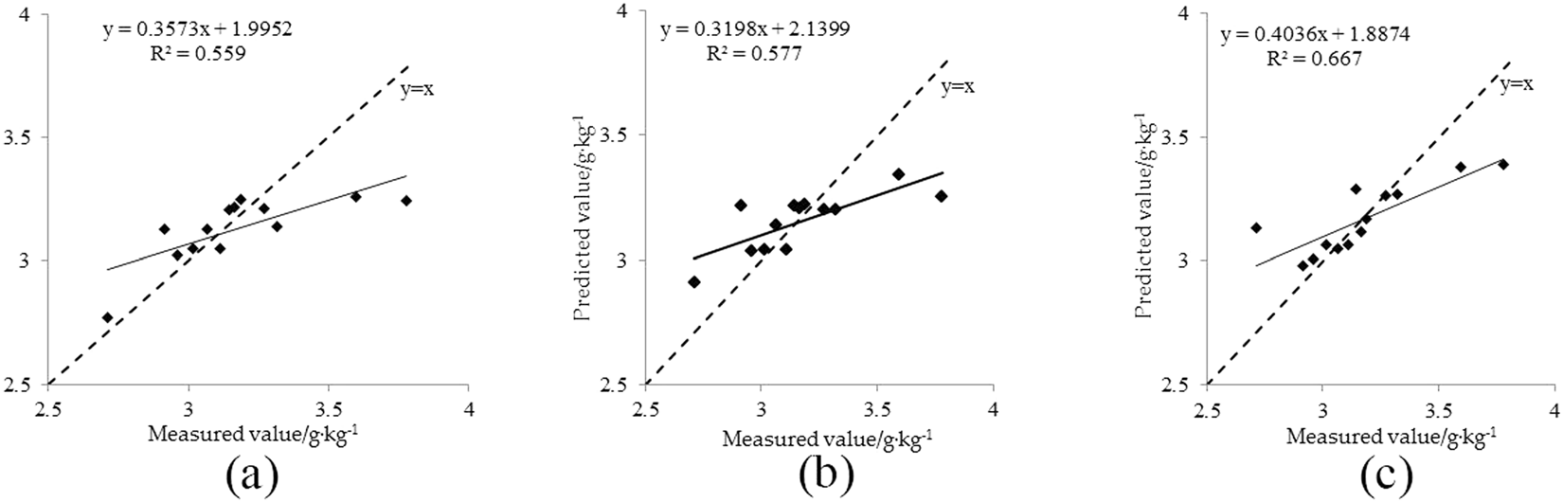
Scatter plots of the measured and predicted values of validation with SVMR 1(**a**), 2(**b**) and 3(**c**) based on vegetation indices.

The surface albedo and chlorophyll content of 13 samples were used to test the model. As shown in Table 8, the verification determinant coefficient of model 1 (R_SVMR1v_^2^) was 0.559, and the verification root mean square error (RMSE_SVMR1v_) was 0.197. The verification determinant coefficient of model 2 (R_SVMR2v_^2^) was 0.577, and the verification root mean square error (RMSE_SVMR2v_) was 0.194. The verification determinant coefficient of model 3 (R_SVMR3v_^2^) was 0.667, and the verification root mean square error (RMSE_SVMR3v_) was 0.178. At the same time, the predicted values of the chlorophyll content of the canopy for the tested samples were compared 1:1 with the measured values (e.g., Fig. 6). The determination coefficient of building and testing the SVMR model 3 based on the vegetation indices series 3 (NDVI_green_, NDVI_red_ and NDVI_re_) was maximal, and its RMSE was minimal. This indicates that model 3 has better self-estimation and estimation abilities and can be used for the quantitative estimation of the apple canopy chlorophyll content.

#### Optimization of two estimation models

Comparing the BPNN and the SVMR, in general, the SVMR can have better accuracy than that of the BPNN model because of its structure risk minimization (SRM) principle (whose aim is that of minimizing the upper bound of the generalization error rather than the training error), SVMR has fewer free parameters to optimize and it can eliminate over-fitting training and local minima^31^.

The results supported the opinion above. In the BPNN estimation models, the R^2^ of building and testing for model 3 based on series 3 (NDVI_green_, NDVI_red_ and NDVI_re_) were maximal, and were 0.674 and 0.601, respectively. The RMSE of building and testing were minimal, which were 0.169 and 0.185, respectively. In the SVMR models, the R^2^ of building and testing for model 3 based on series 3 (NDVI_green_, NDVI_red_ and NDVI_re_) are the same as the BPNN model and maximal and were 0.729 and 0.667, respectively. The RMSE of building and testing were minimal, which were 0.159 and 0.178, respectively. Compared with these two models, the SVMR estimation model was more accurate and stabler than the BPNN model, which could be used to retrieve the canopy chlorophyll content of apple trees.

## Conclusions

### The main results are as follows

(1) Atmospheric correction of Sentinel-2A multispectral remote sensing images was carried out. Based on this, the Minnaert model was used to calibrate the image of study area. The Minnaert model could remove the shadow of the terrain, reduce the contrast ratio of the sunny and shade slope, eliminate the effect of terrain, and get the surface reflectivity. The relative error of band 2~8 A was 29.5%, 19.4%, 32.3%, 16.1%, 11.1%, 10.6%, 9.7% and 9.0%, respectively. It showed that more real canopy spectra had been obtained by various image processing.

(2) Considering the spectral characteristics of the green vegetation and the bands of the Sentinel-2A image, 12 vegetation indices were constructed based on the blue band 2, green band 3, red band 4, red edge band 7, near-infrared band 8 and near infrared band 8 A of the Sentinel-2A image, using the principle of RVI, CI and NDVI for reference. 3 vegetation indices series were selected, through the correlation analysis between vegetation index and chlorophyll content, and the autocorrelation analysis of vegetation indices. The three vegetation indices series were series 1 (RVI_blue_, RVI_red_ and RVI_re_), series 2 (CI_blue_, CI_red_ and CI_re_) and series 3 (NDVI_green_, NDVI_red_ and NDVI_re_).

(3) In the BPNN estimation models, the determinant coefficient of building and testing for model 3 based on series 3 (NDVI_green_, NDVI_red_ and NDVI_re_) were maximal and were 0.674 and 0.601, respectively. The RMSE of building and testing of it were minimal, which were 0.169 and 0.185, respectively. In the SVMR models, the determinant coefficient of building and testing for model 3 based on series 3 (NDVI_green_, NDVI_red_ and NDVI_re_) same as the BPNN model were maximal and were 0.729 and 0.667, respectively. The RMSE of building and testing of it were minimal, which were 0.159 and 0.178, respectively. Compared with these two models, the SVMR estimation model was better than the BPNN model, which could be used to retrieve the canopy chlorophyll content of apple trees.

### Data availability statement

The experimental data were measured according to the test specifications, which can be used for further analysis.
